# Case Report: Two cases of advanced primary cardiac angiosarcoma treated with anlotinib and a retrospective analysis of the literature

**DOI:** 10.3389/fcvm.2024.1363235

**Published:** 2024-03-22

**Authors:** Pan Yang, Fu Xiong, Bingjing Zhu, Liang Gong, Chunlan Tang

**Affiliations:** Department of Respiratory and Critical Care Medicine, First Affiliated Hospital of Army Medical University, Chongqing, China

**Keywords:** cardiac angiosarcoma, pleural effusion, pericardial effusion, vascular targeting agents, anlotinib

## Abstract

Primary cardiac angiosarcoma is a rare malignant soft-tissue sarcoma derived from vascular endothelial cells or lymphatic endothelial cells, with a high malignancy, poor prognosis, and a lack of effective medical therapy. This article reports on two patients with primary cardiac angiosarcoma who received first-line treatment with multi-targeted anti-angiogenic agent, anlotinib monotherapy. The treatment rapidly controlled pleural and pericardial effusion, significantly reduced the tumor, improved symptoms, and showed satisfactory recent efficacy. This indicates that anlotinib offers a new first-line treatment option for advanced primary cardiac angiosarcoma.

## Introduction

Primary cardiac angiosarcoma (PCA) is a rare malignant tumor originating from vascular endothelial cells or lymphatic endothelial cells, but it accounts for one-third to one-half of all cardiac sarcomas (1, 2). It usually occurs in the right atrium, but also in the right ventricle and pericardium. There is no gender difference in incidence rate. Most patients are between 40 and 59 years old at the time of diagnosis. They are highly invasive and can quickly invade adjacent structures. 47%–89% of patients have lung, liver or brain metastasis at the time of initial diagnosis (3, 4). Its prognosis is poor, with a median PFS of about 5 months and a median OS of about 12 months. Over 90% of patients experience metastasis or local recurrence within 2 years, and the 2-year survival rate is less than 30% (5). The clinical manifestations are not specific and largely depend on the location and extent of the tumor. Surgery is the primary treatment for PCA, followed by radiotherapy and/or chemotherapy, but the effects and effectiveness of radiotherapy and chemotherapy still require comprehensive study (6). Currently, traditional treatment methods have limited benefits for patients, and it is crucial to discover effective treatment approaches. Targeted therapy has emerged as a current research focus. Presently, research has confirmed that tumor-targeting drugs such as sorafenib, sunitinib and pazopanib have certain therapeutic effects on angiosarcoma (7–9). However, due to the limited number of cases, designing clinical trials is challenging, and large-scale randomized controlled clinical trials are still lacking, resulting in the absence of a standard treatment plan. Anlotinib, a multi-target tyrosine kinase inhibitor, has inhibitory effects on targets such as VEGFR1, VEGFR2, VEGFR3, PDGFR, FGFR, etc., which is beneficial for controlling tumor invasion and metastasis. Additionally, anlotinib also acts on C-KIT targets and can inhibit the growth of tumor cells (10). The results of the ALTER0203 study demonstrated that compared to the control group, anlotinib significantly prolonged the median PFS in various subtypes of sarcoma (6.27 months vs. 1.47 months) and significantly improved the objective response rate (10.13% vs. 1.33%) and disease control rate (55.7% vs. 22.67%). Based on this, it has been approved by NMPA for monotherapy in patients with acinar soft tissue sarcoma, clear cell sarcoma, and other advanced soft tissue sarcoma who have previously received anthracycline-containing treatment. Our department admitted two cases of primary cardiac angiosarcoma. One case was primary in the pericardium, and the other was primary in the right atrium. After receiving monotherapy with anlotinib, satisfactory short-term efficacy was achieved. We will analyze these two cases based on the specific details.

## Case 1 presentation

**Figure 1:**
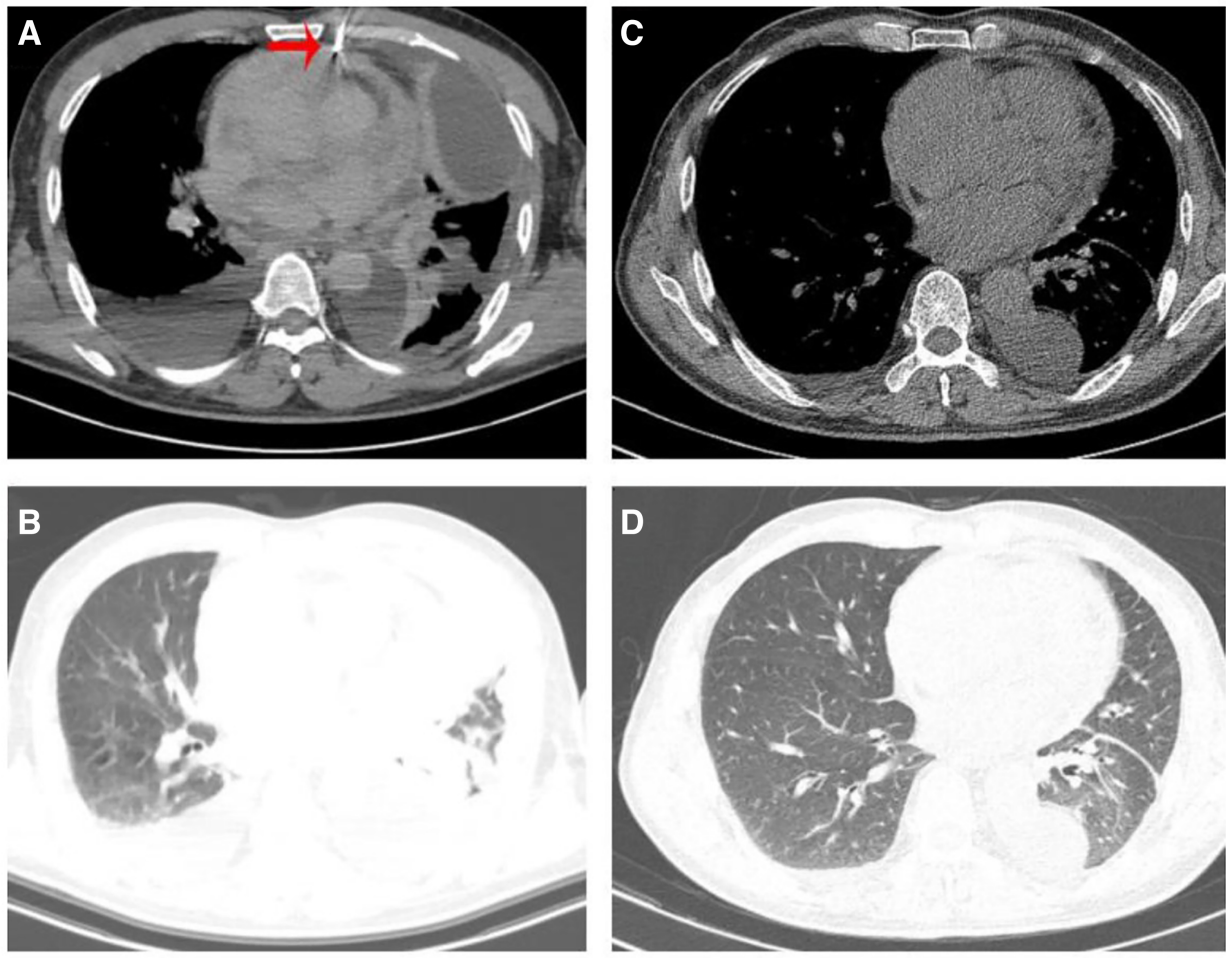
(**A**) Patient underwent CT guided pericardial biopsy. (**A,B**) Chest CT scan revealed bilateral pleural effusion, pericardial thickening, and pericardial effusion before treatment. (**C,D**) Chest CT showed a significance reduction in pleural and pericardial effusion after receiving one cycle of anlotinib treatment.

**Figure 2:**
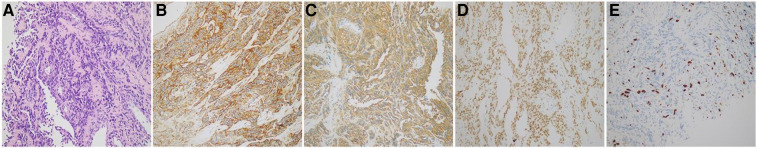
(**A**) Tumor cell staining of pericardial tissue (HE staining, ×20). (**B–E**) Staining of tumor markers in cellular tissues (immunohistochemical, ×20). (**B**) CD31 protein expression; (**C**) D2-40 protein expression; (**D**) ERG protein expression; (**E**) Ki-67 protein expression. Immunohistochemical results showed that D2-40 and ERG in tumor cells were diffusely positive; Ki-67 positive rate was about 30%; and CD31 in tumor cells were diffusely strongly positive.

A 64-year-old Chinese male presented with chest pain, shortness of breath, edema in both lower limbs, and headaches at our hospital. He is a smoker with a smoking index of 20 pack years. Thoracic fluid B-ultrasound revealed bilateral pleural effusion (liquid dark areas with a distance of approximately 38 mm detected in the right chest, and liquid dark areas with a distance of approximately 107 mm detected in the left chest). Left chest closed drainage was performed, and the pleural fluid analysis showed bloody turbidity, a positive Li Fanta test, a total cell count of 191.824 × 10^9^/L, a white blood cell count of 0.678 × 10^9^/L, a monocyte percentage of 94%, and a multinucleated cell percentage of 6%. Chest fluid biochemistry revealed glucose levels of 6.04 mmol/L, total protein levels of 36.9 g/L, albumin levels of 27.6 g/L, globulin levels of 9.3 g/L, a white blood cell ratio of 2.97, lactate dehydrogenase levels of 386 IU/L, and adenylate dehydrogenase levels of 1.68 U/L. Chest enhanced CT scan showed a visible size of approximately 7.3 cm at the upper edge of the right atrium and a 5.7 cm irregular mass shadow with an unclear boundary adjacent to the right atrium and superior vena cava. The lesion exhibited significantly uneven enhancement on enhanced scans and closely adhered to the pericardium, causing significant uneven cystic enhancement and a small amount of pericardial effusion. Bilateral pleural effusion was observed, partially enveloped on the left, with thickening of the left pleura and irregular cystic enhancement of the anterior and lower pleura (Figures 1A,B). PET/CT scan indicated irregular thickening of the pericardium and left pleura, increased FDG metabolism, and a high possibility of malignancy. A large amount of pleural effusion was present on the left side. Biopsy of the left pleura (in the 8th intercostal space of the left scapular line) suggested a high possibility of a vascular tumor, epithelioid endothelial cell tumor, or angiosarcoma in the left pleura. Immunohistochemical results showed CK (–), Vim (+), CEA (+), WT-1 (+), TTF-1 (–), P63 (–), Ki-67 (+, 30%), Napsina (–), CR (–), ERG (+), CD31 (+), CD45 (–), Desmin (–). To further clarify the diagnosis, a CT-guided pericardial biopsy was performed again, indicating that pericardial angiosarcoma had immunohistochemical features such as CK (–), TTF-1 (–), CR (–), D2-40 (+), P53 (–), Ki-67 (+, 10%), ERG (+), CD31 (+), HHV-8 (−), Desmin (−) (Figures 2A–E). Based on imaging and pathological diagnosis, it was determined to be a primary cardiac angiosarcoma with pleural metastasis (cTxN0M1, stage IV). The patient declined systemic chemotherapy. Approximately 700–1,000 ml of pleural effusion was drained daily. After 3 days of treatment with anlotinib, the patient's pleural effusion significantly decreased, and shortness of breath improved. Following one cycle of treatment (anlotinib, 12 mg oral/day, days 1–14, 21 days per cycle, 1cycle), the patient's symptoms significantly improved. Chest CT examination showed that the space-occupying lesion at the upper edge of the right atrium was smaller than before, and there was a significant decrease in pericardial and right pleural effusion (Figures 1C,D). The recent therapeutic effect was satisfactory. But due to economic reasons, the patient used anlotinib for only one treatment cycle and did not continue taking anlotinib orally. Subsequently, the patient's condition worsened, and the overall OS is five months.

## Case 2 presentation

**Figure 3:**
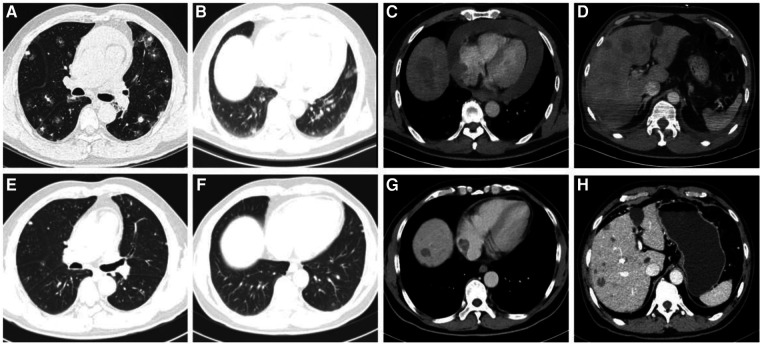
(**A–D**) Chest CT scan of the patient revealed dispersed sub-solid ground glass shadows in both lungs, soft tissue shadows in the right atrium, numerous nodular shadows in the liver, and a considerable pericardial effusion before treatment. (**E,F**) After 4 cycles of anlotinib treatment, the patient's numerous nodules in the lungs and liver showed significant reduction, the tumor lesion in the right atrium decreased, and pericardial effusion as managed.

A 57-year-old Chinese male presented to our hospital with the main complaint of discovering bilateral pulmonary nodules six months ago and experiencing shortness of breath for the past three months. He has a pre-existing condition of hyperemia and is a heavy smoker with a smoking index of 40 pack years. On November 5, 2022, a physical examination revealed multiple scattered sub-solid ground glass shadows in both lungs, with a maximum length of approximately 25 mm. At that time, the patient did not experience any discomfort. After three months (February 2023), the patient developed shortness of breath after physical activity, which could be relieved by rest, but the patient did not pay attention to it. The shortness of breath gradually worsened with activity, and there was no improvement after receiving symptomatic treatment at an external hospital. Three months later (May 12, 2023), a chest enhanced CT scan showed larger scattered sub-solid ground glass shadows in both lungs compared to May 11, with a maximum length diameter of about 35 mm. Additionally, soft tissue density shadows were found in the right atrium, along with a small amount of pericardial effusion (Figures 3A–D). A PET/CT scan on May 16 revealed a large amount of fluid accumulation in the pericardial cavity, with a maximum diameter of about 1.8 cm. There was an increase in the arc-shaped soft tissue density around the root of the superior vena cava and the right atrium, accompanied by a significant increase in FDG uptake. The boundary with the adjacent pericardium and atrial wall was unclear, with SUVmax at 13.1. Multiple slightly lower density nodules of varying sizes were observed in the right lobe of the liver, with larger ones located in the parietal area of the liver, measuring approximately 2.2 cm in diameter with unclear boundaries. FDG uptake was slightly increased, with SUVmax at 4.4. Solid nodular shadows were scattered in both lungs, with visible halo signs. FDG uptake was slightly increased, with SUVmax at 2.1.

**Figure 4:**
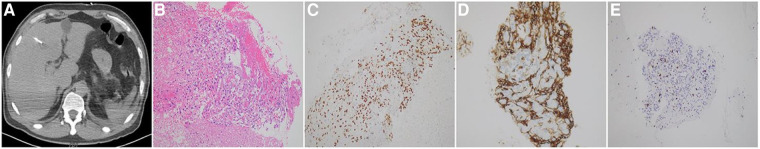
(**A**) CT guided liver biopsy. (**B**) Tumor cell staining of liver tissue (HE staining, ×20). (**C–E**) Tumor marker staining of liver cell tissue (immunohistochemical, ×20); (**C**) CD31 protein expression; (**D**) ERG protein expression; (**E**) Ki-67 protein expression. Immunohistochemical results showed that CD31 and ERG in tumor cells were diffusely positive; Ki-67 positive rate was about 10%.

The patient was admitted to our hospital for treatment from May 5 to May 28. On May 29, a cardiac ultrasound revealed low echogenicity in the right atrium (visible range approximately 47 mm × 32 mm low echo), mild regurgitation of the mitral, tricuspid, and pulmonary valves, and moderate to large amounts of pericardial effusion (liquid dark areas with a spacing of 9 mm detected in the posterior left ventricular wall, liquid dark areas with a spacing of 18 mm detected in the anterior right ventricular wall, liquid dark areas with a spacing of 18 mm detected in the apex of the heart, liquid dark areas with a spacing of 19 mm detected in the lateral left ventricular wall, liquid dark areas with a spacing of 19 mm detected in the lateral right ventricular wall, and liquid dark areas with a spacing of 13 mm detected in the right atrium and lower right ventricle). A closed drainage of the pericardial cavity was performed, and routine analysis of the pericardial effusion showed bloody turbidity, positive Li Fanta test, total number of cells at 2,345.962 × 10^9^/L, white blood cell count at 1.863 × 10^9^/L, monocyte percentage at 68%, and multinucleated cell percentage at 32%. Chest fluid biochemistry: glucose level of 4.13 mmol/L, total protein level of 60.4 g/L, albumin level of 33.7 g/L, globulin level of 26.7 g/L, white blood cell ratio of 1.26, lactate dehydrogenase level of 262.80IU/L, adenylate dehydrogenase level of 14.6U/L. Liver enzyme display: ALT level of 1,487.2 IU/L, AST level of 1,452.9 IU/L, ALP level of 183 IU/L, daily drainage of approximately 500–700 ml of hemorrhagic pericardial effusion. Following admission, a lung biopsy revealed left lung alveolar epithelial and interstitial hyperplasia, mucinous degeneration, deposition of hemosiderin, and chronic tissue inflammation. Two exfoliative cytological examinations of the pericardial effusion showed the presence of a few inflammatory cells and mesothelial cells in the blood clot. To confirm the diagnosis, a liver puncture biopsy was performed (Figure 4A), which indicated angiosarcoma; CK (–), CD34 (stove+), P53 (2%+), Ki-67 (+, 10%), ERG (+), CD31 (+), S-100 (–), HMB45 (–), Desmin (–), SMA (stove+) (Figures 4B–E). Blood gene test results showed no clinically significant mutations, TMB: 6.8 mutations/Mb, and no detection of MSI-H. Based on imaging examination and pathological diagnosis, it was determined to be a primary cardiac angiosarcoma with bilateral lung and liver metastasis (cT1N0M1, stage IV). Starting from June 16, 2023, the patient received targeted treatment with anlotinib (anlotinib, 12 mg oral/day, days 1–14, 21 days per cycle, 11cycle). By June 18, 2023, the pericardial drainage fluid gradually decreased in volume and became lighter in color. After extracting 100 ml of pericardial effusion, no further extraction was necessary. Following the treatment, the patient experienced significant relief from shortness of breath, and their transaminase levels returned to normal. Subsequent CT scans revealed a significant reduction in tumor lesions in the right atrium, liver, and lungs, and the pericardial effusion was under control. No noticeable side effects from the medication were observed (Figures 3E–H). As of February 27, 2024, the patient has undergone 11 cycles of anlotinib treatment, resulting in partial remission with no notable drug side effects, PFS exceeding eight months.

## Discussion

Among all cardiac tumors in adults, approximately 25% are malignant, with primary cardiac sarcoma representing around 95% of all malignant cardiac tumors. Notably, vascular sarcoma is the most frequently occurring type (11, 12). The key characteristic of PCA is its invasive growth and metastasis, frequently affecting the valve, pericardium, and even the coronary artery locally, or spreading throughout the body via the bloodstream (13). Early diagnosis of this condition is challenging, as its clinical symptoms are not typical. It often presents with manifestations such as chest tightness, shortness of breath, arrhythmia, or distant metastasis (14). The main clinical presentation of the two cases we reported is dyspnea, which is linked to tumor infiltration of the pericardium and pleura, resulting in pleural effusion and pericardial effusion. Early detection is challenging, thus imaging examinations hold significant diagnostic value for such patients. Imaging studies are crucial for diagnosing patients with cardiac sarcomas, with transthoracic echocardiography, chest CT, and MRI being commonly used methods. Whole body PET/CT is typically used to identify metastatic sites, while the gold standard for diagnosis is biopsy of the primary or metastatic site. Immunohistochemical markers like CD31, CD34, ERG, and Ki-67 are commonly used for angiosarcoma diagnosis. In our two patients, immunohistochemistry showed positive expression of CD31, ERG, and Ki-67, leading to a diagnosis of primary cardiac angiosarcoma based on imaging and pathology.

Surgical resection is not only the primary treatment for early or limited stage PCA, but also a predictive factor for patient survival (2).

Currently, for late-stage PCA patients who are ineligible for surgery, the treatment plan primarily involves the soft tissue sarcoma treatment plan. The most frequently prescribed medications are anthracyclines or paclitaxel drugs. First-line chemotherapy utilizing anthracyclines has shown remarkable tumor reduction in 25% of patients, with a median progression-free survival (PFS) of 4.9 months and a median overall survival (OS) of 9.9 months (15). In 2021, the Asian Sarcoma Consortium conducted a retrospective analysis of 276 patients with advanced angiosarcoma at 8 sarcoma academic centers. They compared the efficacy of first-line treatment regimens: paclitaxel (47.6% of patients) and liposome doxorubicin (19.6% of patients). The results revealed that paclitaxel monotherapy had a median PFS of 4.5 months, while liposome doxorubicin chemotherapy had a median PFS of 2.8 months. The median OS was 11.9 months for paclitaxel and 10.6 months for liposome doxorubicin. However, the difference between the two treatments was not statistically significant (16). Another phase III randomized controlled trial (EORTC 62012) examined the effectiveness of combination chemotherapy. The study revealed that while the doxorubicin combined with ifosfamide group had a significantly higher overall effective rate compared to the doxorubicin group (26% vs. 14%, *P* < 0.0006), there was no statistically significant difference in overall survival (OS). This lack of significance may be attributed to the heightened toxicity associated with the combination therapy group (17). There was no significant difference in median progression-free survival (PFS) between the doxorubicin and gemcitabine combined with docetaxel groups (23.3 weeks vs. 23.7 weeks). Although this study did not find any advantages of combining gemcitabine with docetaxel as a first-line treatment option (18), it does offer new treatment possibilities for patients with cardiac dysfunction or PCA. Due to the cumulative cardiac toxicity of doxorubicin and cyclophosphamide, the current treatment options for cardiac angiosarcoma mainly involve combining doxorubicin and cyclophosphamide with paclitaxel, docetaxel, or gemcitabine (19–23). However, the median OS for late-stage PCA patients does not surpass 8 months. Nevertheless, because PCA is rare and mostly documented as a case study, there is currently no established treatment method for late-stage cardiac angiosarcoma. It is crucial to investigate new treatment methods and strategies.

Tumor cell growth, invasion, and metastasis are reliant on the tumor microenvironment (TME), which includes tumor blood vessels. These vessels are frequently distorted and dysfunctional, with varying vascular permeability, excessive proliferation, and loss of wall hierarchy. This abnormal vascular system promotes tumor growth, metastasis, and treatment resistance, while selectively blocking the infiltration of certain immune cell types, such as cytotoxic T cells (24, 25). Vascular endothelial growth factor (VEGF) is a crucial factor in tumor angiogenesis (26, 27). For most solid tumors, such as sarcomas, targeted drugs that inhibit tumor angiogenesis primarily focus on VEGF and VEGFR. These drugs work by blocking the formation of new blood vessels, thus impeding the blood supply to the tumor. Additionally, they normalize the tumor blood vessels, which helps in suppressing tumor growth, development, and metastasis. Moreover, they enhance the response to other treatments (28). Typically, it is frequently combined with chemotherapy, immunotherapy, and radiation therapy for cancer treatment.

Angiosarcoma, a malignant tumor originating from vascular or lymphatic endothelial cells, may have unique advantages in anti-tumor angiogenesis. Ren et al. reported a case of a 99-year-old Chinese man diagnosed with angiosarcoma on his head and face. Due to his advanced age and extensive lesions, chemotherapy options were limited and surgery was challenging. As a result, he received monotherapy with anlotinib for 10 months (12 mg/day, D1-14, 21 days per cycle). The lesions on his head and face significantly decreased, and he tolerated the adverse drug reactions well (29). Both of our cardiac angiosarcoma patients received anlotinib treatment for approximately 3 days, leading to a notable decrease in the rapid expansion of pleural and pericardial effusion, as well as swift relief from symptoms like breathing difficulties. This outcome is attributed to anlotinib's ability to impede blood supply, normalize tumor blood vessels, and reduce vascular permeability. Subsequent assessments revealed partial remission of the target lesion in a short timeframe, which is linked to anlotinib's inhibition of the C-kit gene, thereby impeding tumor cell growth. Neither patient experienced significant toxic side effects, and their quality of life notably improved. In 2019, Case 1 was treated with anlotinib at a monthly expense of 10,227 RMB. Currently, anlotinib is available in hospitals at a monthly cost of 5,760 RMB, a notable reduction from the 2019 price. Anlotinib also offers a free medication policy. After self-paying for 9 courses of anlotinib, patients can receive free medication until disease progression. Therefore, anlotinib could be considered a novel first-line treatment choice, either as a standalone therapy or in combination, for patients with advanced cardiac angiosarcoma presenting with significant pleural or pericardial effusion.

For patients with unresectable angiosarcoma, chemotherapy and radiotherapy are the primary treatment methods, although their efficacy is limited. However, they still provide clinical benefits to patients. Previously, we investigated the use of anlotinib chemotherapy combined with immunotherapy in other types of sarcomas. In a phase II single-arm prospective study, we explored the use of epirubicin in combination with anlotinib as a first-line treatment for advanced unresectable soft tissue sarcoma. The study showed impressive efficacy, with a median progression-free survival (PFS) of 11.5 months, an objective response rate (ORR) of 13.33%, and a disease control rate (DCR) of 80.0%. The safety profile was also good (30). At the 2023 ASCO conference, mid-term data was presented on the safety and efficacy of combining Penpulimab, anlotinib, and epirubicin as a first-line treatment for soft tissue sarcoma. The results showed a median PFS of 10.55 months, an ORR of 12.50% (4/24), and a DCR of 68.75% (14/24), indicating favorable safety. Therefore, the combination of anlotinib and other treatment methods such as chemotherapy, immunization, and palliative radiotherapy is worth further exploration in the treatment of advanced angiosarcoma, in order to maximize patient survival.

## Data Availability

The original contributions presented in the study are included in the article/Supplementary Material, further inquiries can be directed to the corresponding authors.
